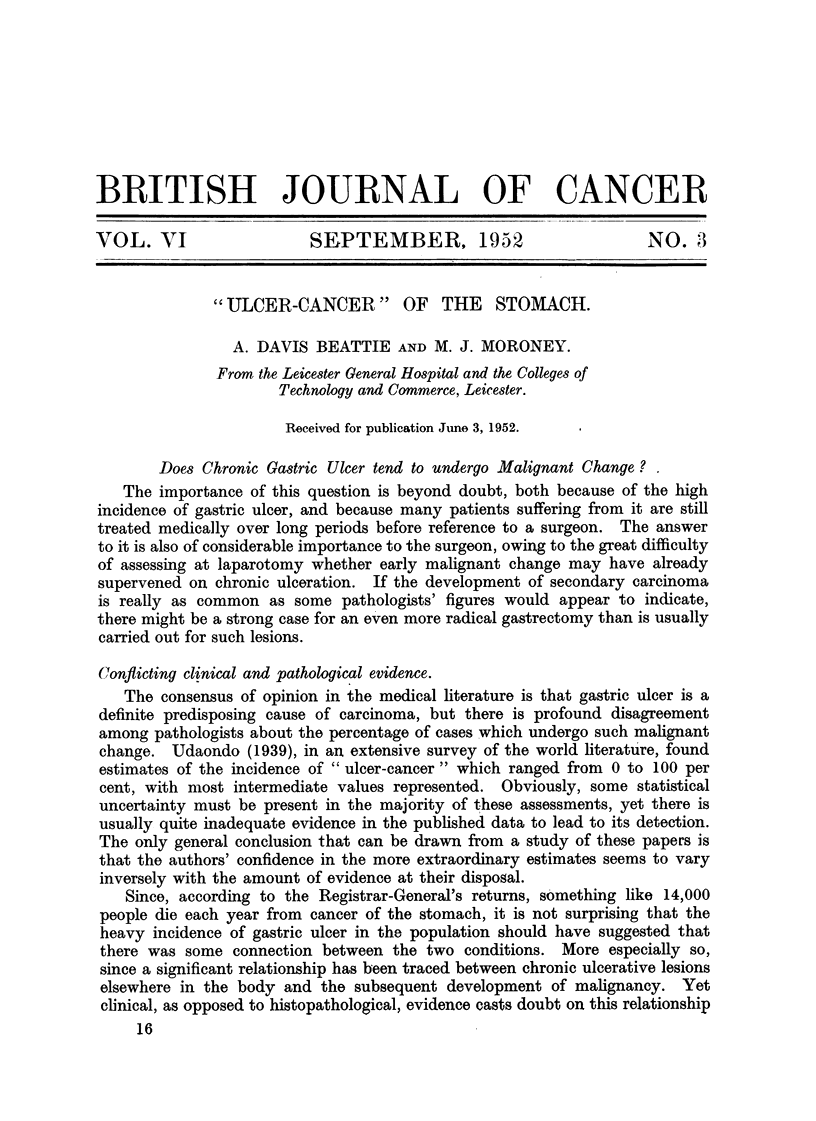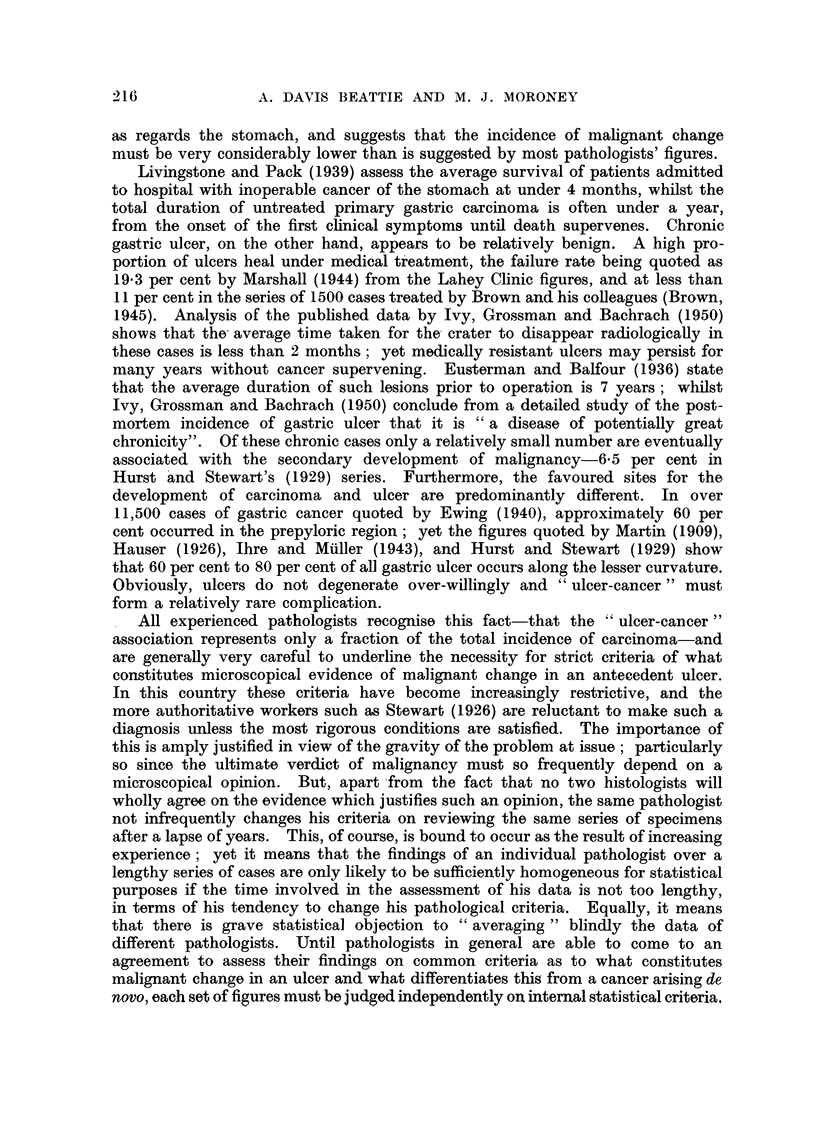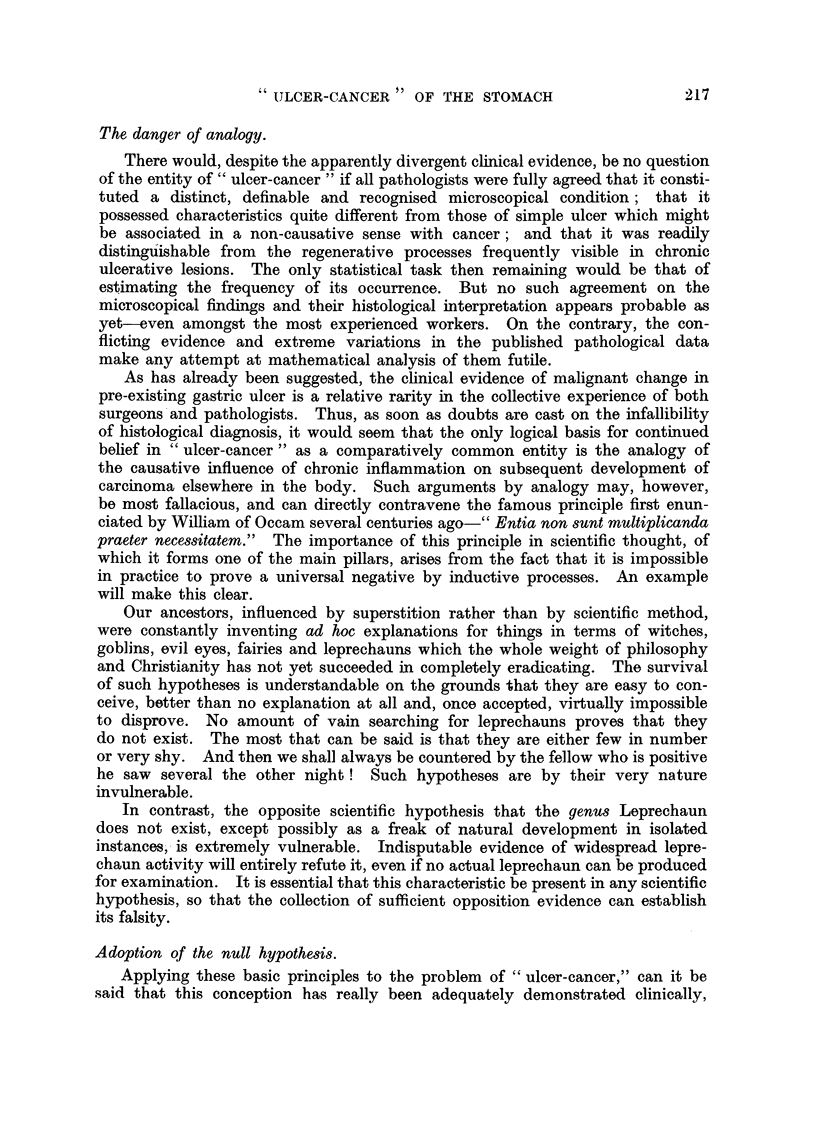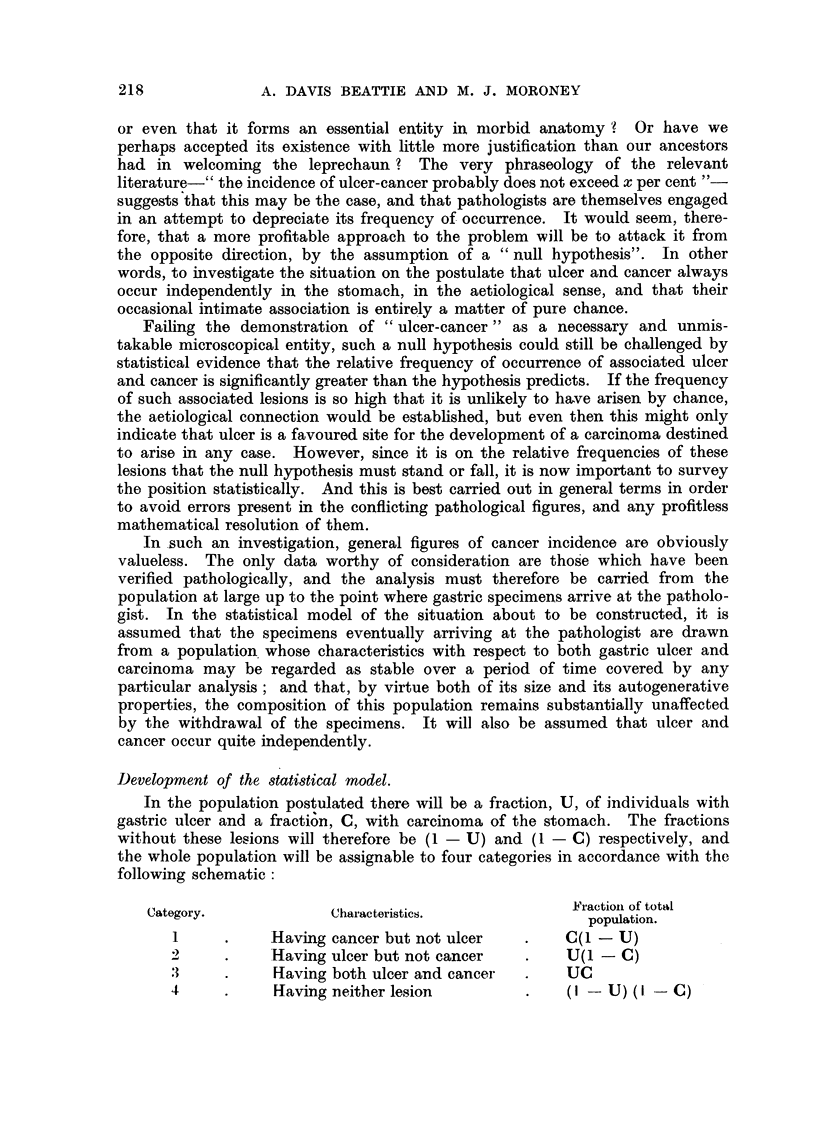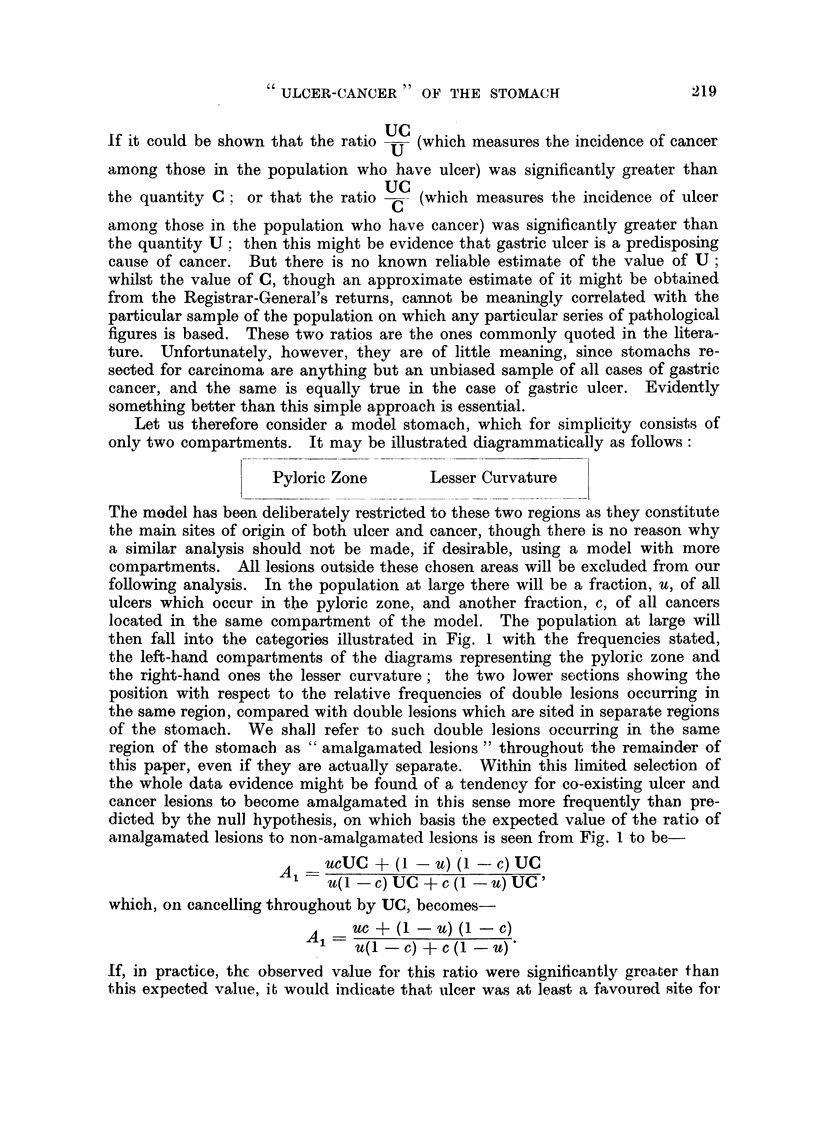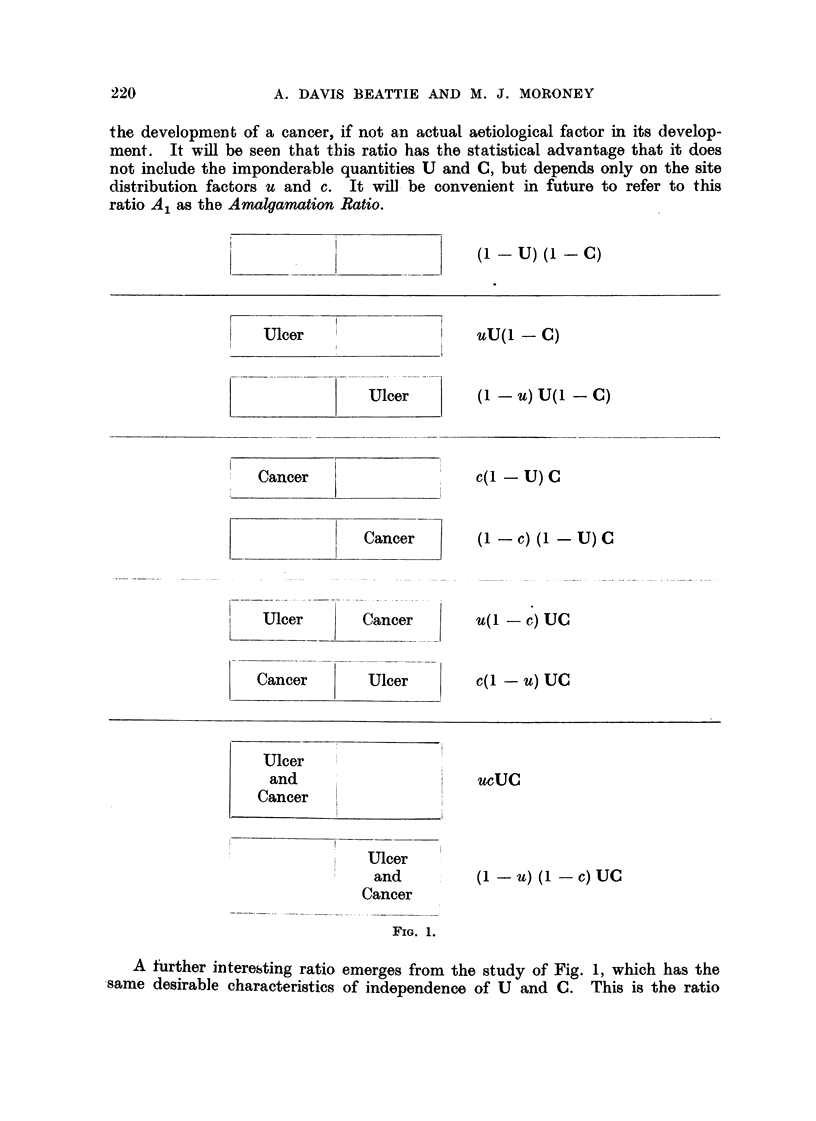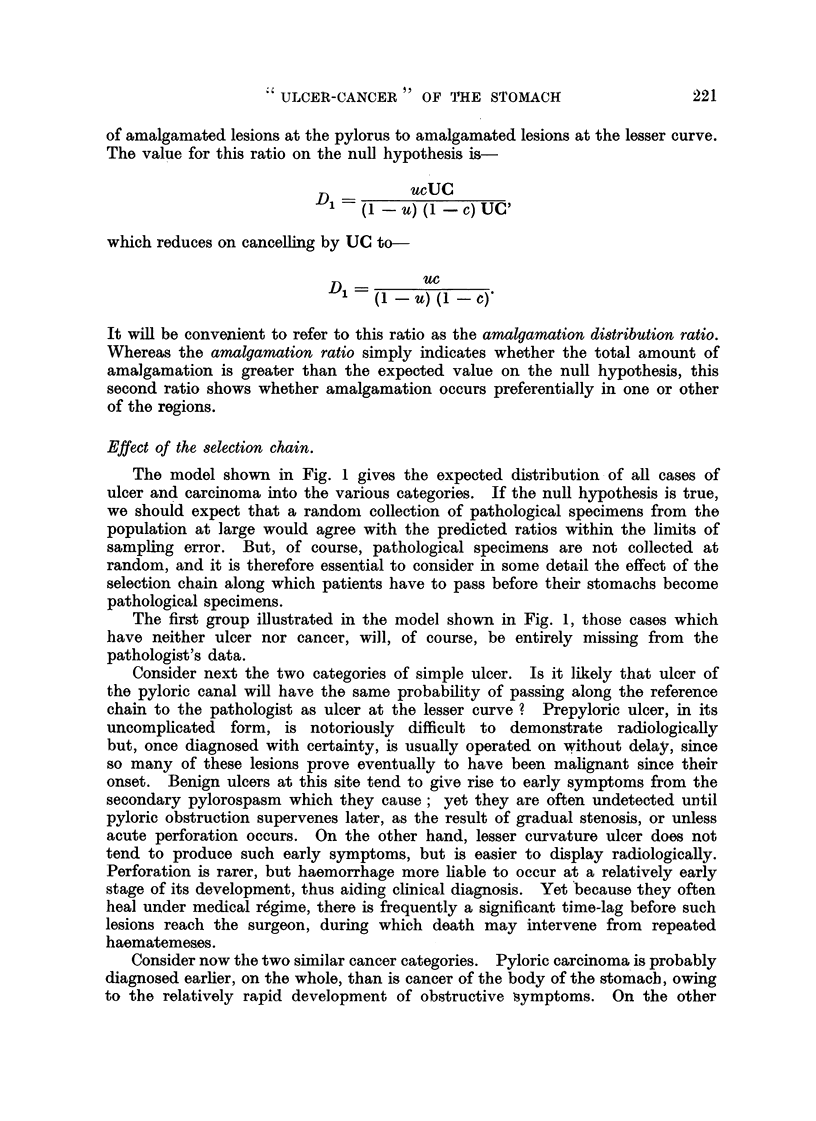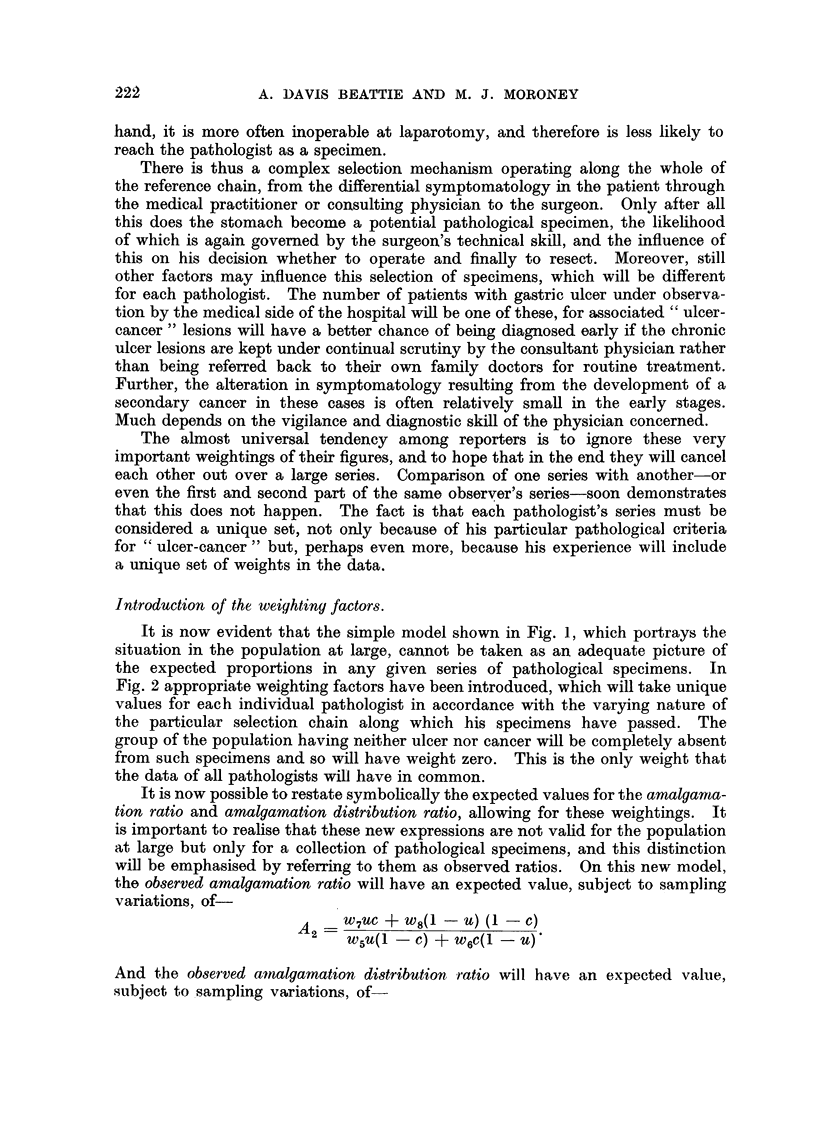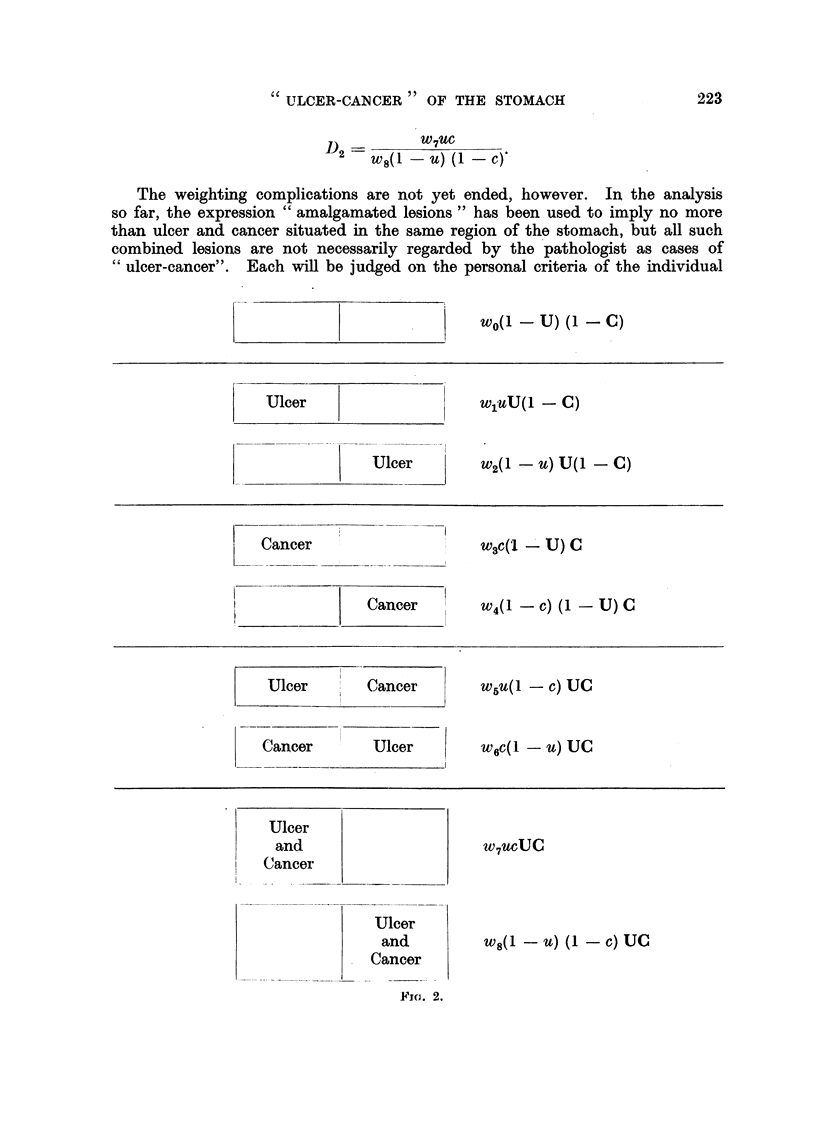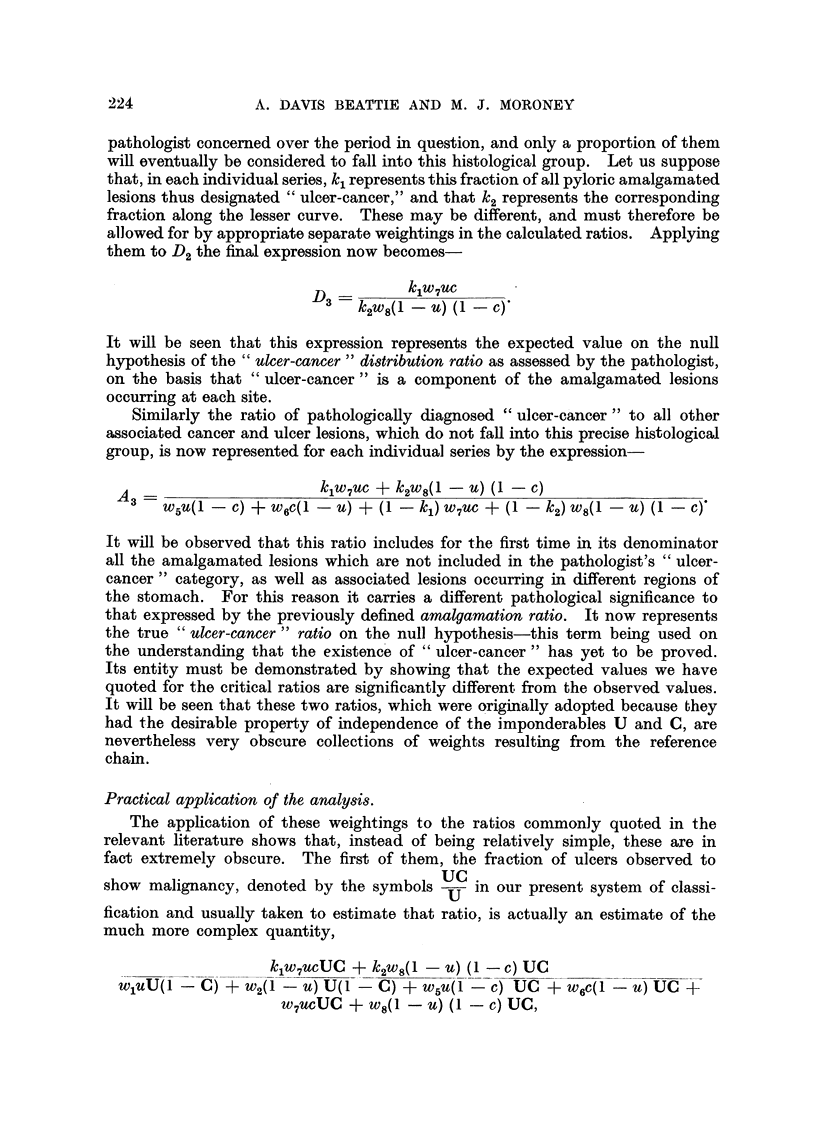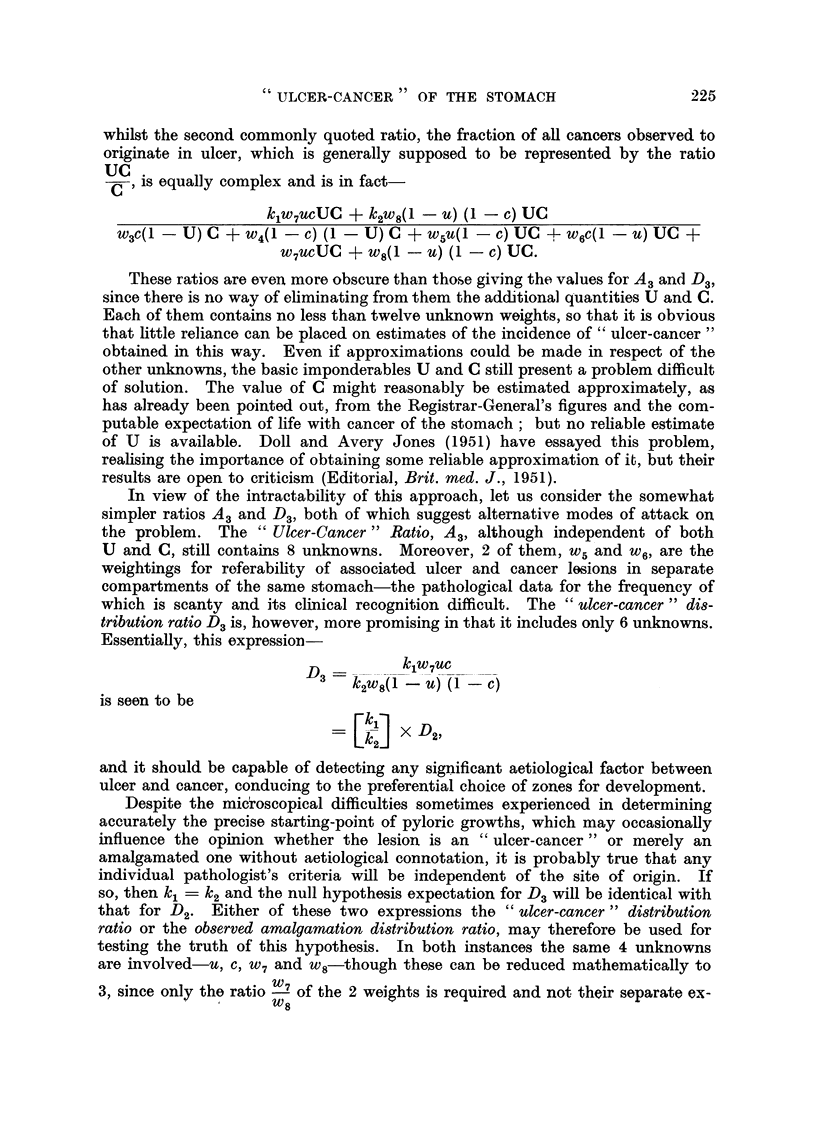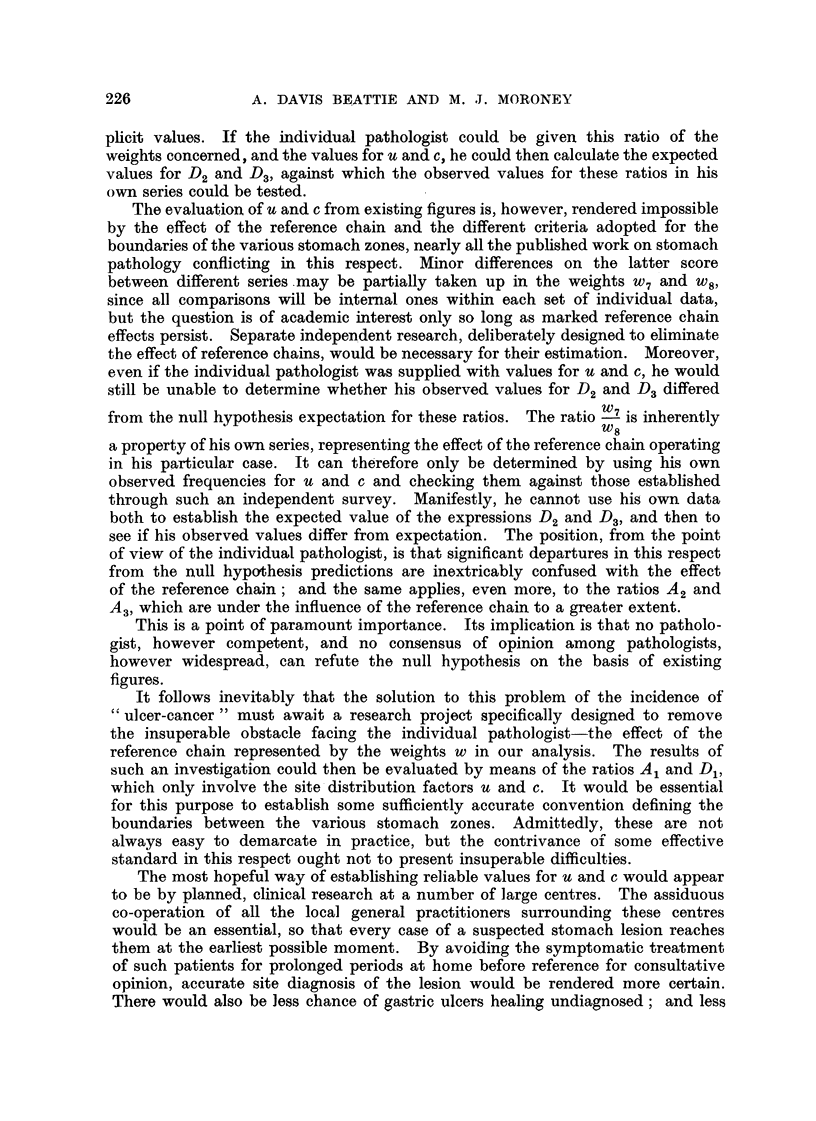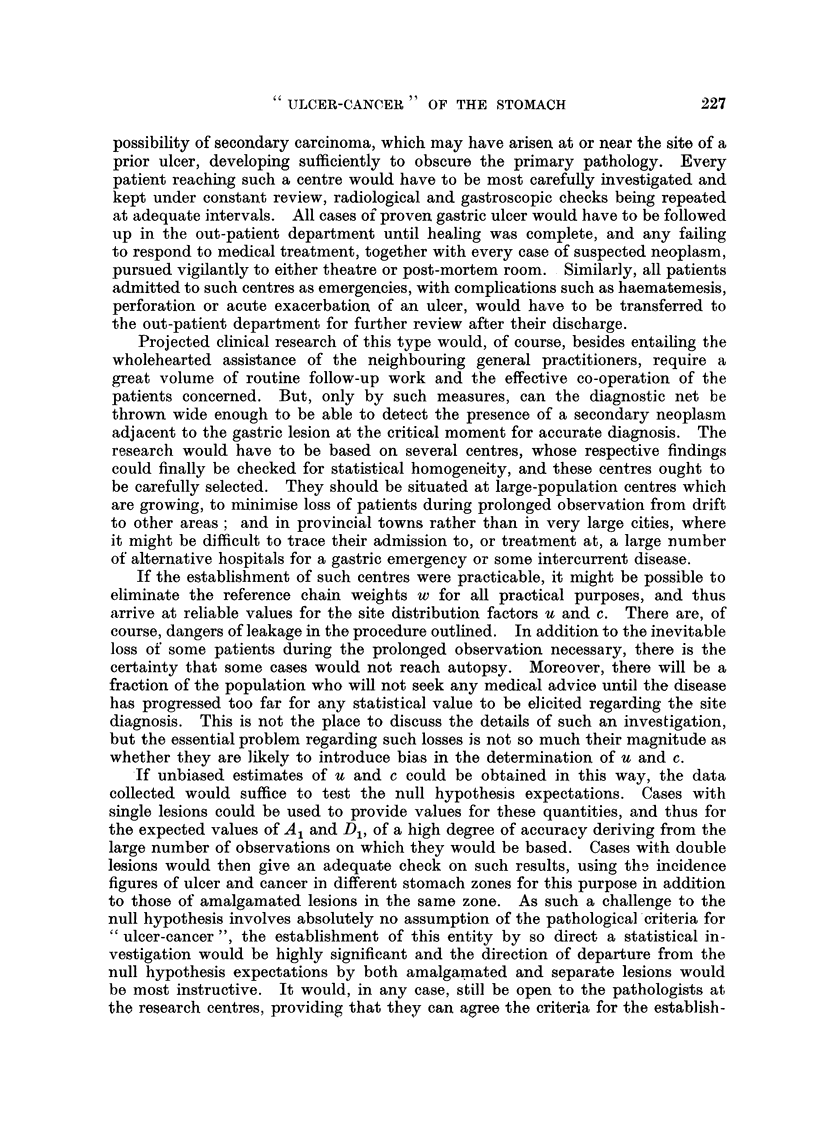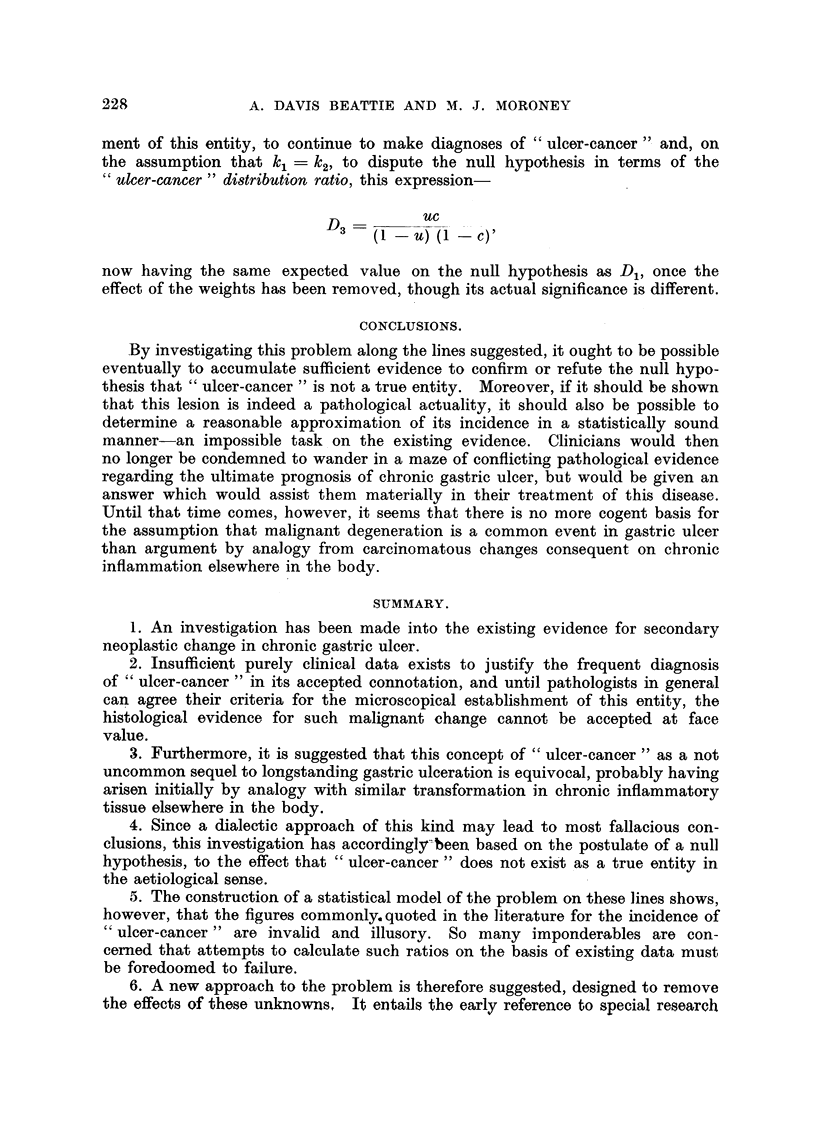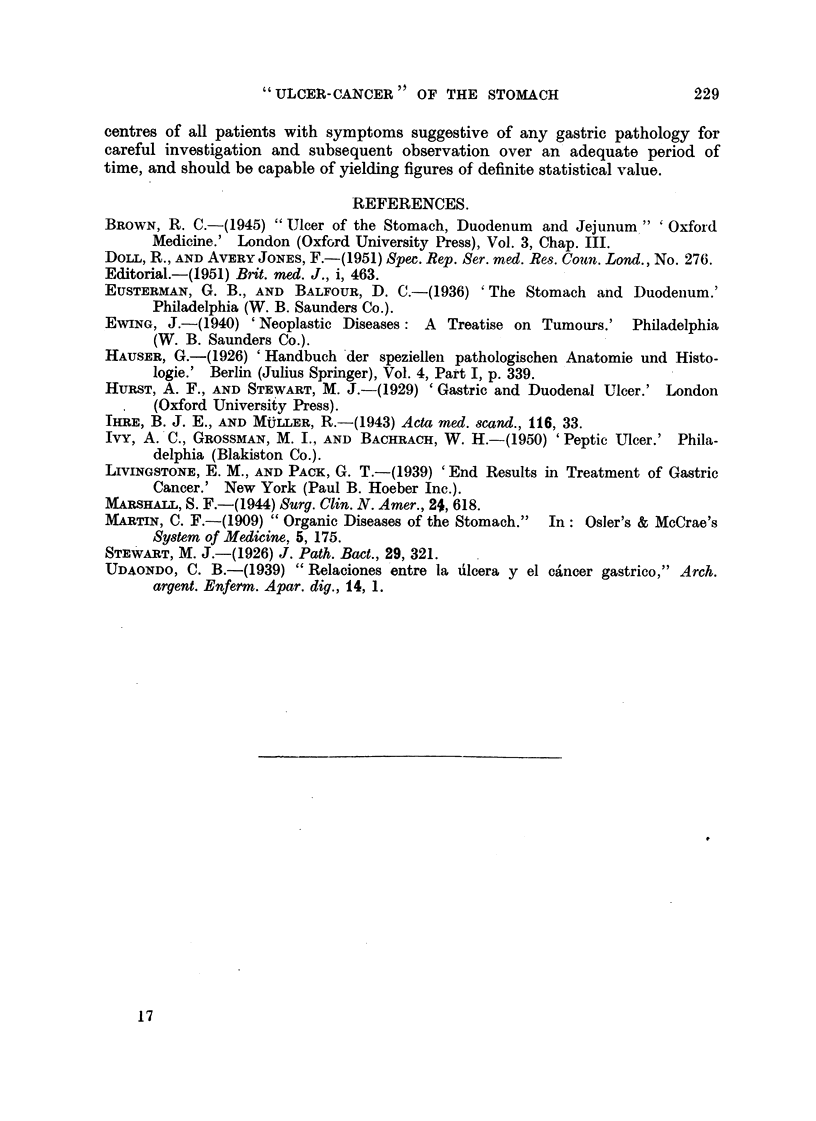# “Ulcer-Cancer” of the Stomach

**DOI:** 10.1038/bjc.1952.24

**Published:** 1952-09

**Authors:** A. Davis Beattie, M. J. Moroney


VOL. VI SEPTEMBER, 1952 NO. 3

### "ULCER-CANCER" OF THE STOMACH.

A. DAVIS BEATTIE AND M. J. MORONEY.

*From the Leicester General Hospital and the Colleges of Technology and Commerce, Leicester.*

Received for publication June 3, 1952.

*Does Chronic Gastric Ulcer tend to undergo Malignant Change?*

The importance of this question is beyond doubt, both because of the high incidence of gastric ulcer, and because many patients suffering from it are still treated medically over long periods before reference to a surgeon. The answer to it is also of considerable importance to the surgeon, owing to the great difficulty of assessing at laparotomy whether early malignant change may have already supervened on chronic ulceration. If the development of secondary carcinoma is really as common as some pathologists' figures would appear to indicate, there might be a strong case for an even more radical gastrectomy than is usually carried out for such lesions.

*Conflicting clinical and pathological evidence.*

The consensus of opinion in the medical literature is that gastric ulcer is a definite predisposing cause of carcinoma, but there is profound disagreement among pathologists about the percentage of cases which undergo such malignant change. Udaondo (1939), in an extensive survey of the world literature, found estimates of the incidence of "ulcer-cancer" which ranged from 0 to 100 per cent, with most intermediate values represented. Obviously, some statistical uncertainty must be present in the majority of these assessments, yet there is usually quite inadequate evidence in the published data to lead to its detection. The only general conclusion that can be drawn from a study of these papers is that the authors' confidence in the more extraordinary estimates seems to vary inversely with the amount of evidence at their disposal.

Since, according to the Registrar-General's returns, something like 14,000 people die each year from cancer of the stomach, it is not surprising that the heavy incidence of gastric ulcer in the population should have suggested that there was some connection between the two conditions. More especially so, since a significant relationship has been traced between chronic ulcerative lesions elsewhere in the body and the subsequent development of malignancy. Yet clinical, as opposed to histopathological, evidence casts doubt on this relationship as regards the stomach, and suggests that the incidence of malignant change must be very considerably lower than is suggested by most pathologists' figures.

Livingstone and Pack (1939) assess the average survival of patients admitted to hospital with inoperable cancer of the stomach at under 4 months, whilst the total duration of untreated primary gastric carcinoma is often under a year, from the onset of the first clinical symptoms until death supervenes. Chronic gastric ulcer, on the other hand, appears to be relatively benign. A high proportion of ulcers heal under medical treatment, the failure rate being quoted as 19·3 per cent by Marshall (1944) from the Lahey Clinic figures, and at less than 11 per cent in the series of 1500 cases treated by Brown and his colleagues (Brown, 1945). Analysis of the published data by Ivy, Grossman and Bachrach (1950) shows that the average time taken for the crater to disappear radiologically in these cases is less than 2 months ; yet medically resistant ulcers may persist for many years without cancer supervening. Eusterman and Balfour (1936) state that the average duration of such lesions prior to operation is 7 years ; whilst Ivy, Grossman and Bachrach (1950) conclude from a detailed study of the post-mortem incidence of gastric ulcer that it is "a disease of potentially great chronicity". Of these chronic cases only a relatively small number are eventually associated with the secondary development of malignancy—6·5 per cent in Hurst and Stewart's (1929) series. Furthermore, the favoured sites for the development of carcinoma and ulcer are predominantly different. In over 11,500 cases of gastric cancer quoted by Ewing (1940), approximately 60 per cent occurred in the prepyloric region ; yet the figures quoted by Martin (1909), Hauser (1926), Ihre and Müller (1943), and Hurst and Stewart (1929) show that 60 per cent to 80 per cent of all gastric ulcer occurs along the lesser curvature. Obviously, ulcers do not degenerate over-willingly and "ulcer-cancer" must form a relatively rare complication.

All experienced pathologists recognise this fact—that the "ulcer-cancer" association represents only a fraction of the total incidence of carcinoma—and are generally very careful to underline the necessity for strict criteria of what constitutes microscopical evidence of malignant change in an antecedent ulcer. In this country these criteria have become increasingly restrictive, and the more authoritative workers such as Stewart (1926) are reluctant to make such a diagnosis unless the most rigorous conditions are satisfied. The importance of this is amply justified in view of the gravity of the problem at issue ; particularly so since the ultimate verdict of malignancy must so frequently depend on a microscopical opinion. But, apart from the fact that no two histologists will wholly agree on the evidence which justifies such an opinion, the same pathologist not infrequently changes his criteria on reviewing the same series of specimens after a lapse of years. This, of course, is bound to occur as the result of increasing experience ; yet it means that the findings of an individual pathologist over a lengthy series of cases are only likely to be sufficiently homogeneous for statistical purposes if the time involved in the assessment of his data is not too lengthy, in terms of his tendency to change his pathological criteria. Equally, it means that there is grave statistical objection to "averaging" blindly the data of different pathologists. Until pathologists in general are able to come to an agreement to assess their findings on common criteria as to what constitutes malignant change in an ulcer and what differentiates this from a cancer arising *de novo*, each set of figures must be judged independently on internal statistical criteria.

*The danger of analogy.*

There would, despite the apparently divergent clinical evidence, be no question of the entity of " ulcer-cancer " if all pathologists were fully agreed that it constituted a distinct, definable and recognised microscopical condition ; that it possessed characteristics quite different from those of simple ulcer which might be associated in a non-causative sense with cancer ; and that it was readily distinguishable from the regenerative processes frequently visible in chronic ulcerative lesions. The only statistical task then remaining would be that of estimating the frequency of its occurrence. But no such agreement on the microscopical findings and their histological interpretation appears probable as yet—even amongst the most experienced workers. On the contrary, the conflicting evidence and extreme variations in the published pathological data make any attempt at mathematical analysis of them futile.

As has already been suggested, the clinical evidence of malignant change in pre-existing gastric ulcer is a relative rarity in the collective experience of both surgeons and pathologists. Thus, as soon as doubts are cast on the infallibility of histological diagnosis, it would seem that the only logical basis for continued belief in " ulcer-cancer " as a comparatively common entity is the analogy of the causative influence of chronic inflammation on subsequent development of carcinoma elsewhere in the body. Such arguments by analogy may, however, be most fallacious, and can directly contravene the famous principle first enunciated by William of Occam several centuries ago—" *Entia non sunt multiplicanda praeter necessitatem.*" The importance of this principle in scientific thought, of which it forms one of the main pillars, arises from the fact that it is impossible in practice to prove a universal negative by inductive processes. An example will make this clear.

Our ancestors, influenced by superstition rather than by scientific method, were constantly inventing *ad hoc* explanations for things in terms of witches, goblins, evil eyes, fairies and leprechauns which the whole weight of philosophy and Christianity has not yet succeeded in completely eradicating. The survival of such hypotheses is understandable on the grounds that they are easy to conceive, better than no explanation at all and, once accepted, virtually impossible to disprove. No amount of vain searching for leprechauns proves that they do not exist. The most that can be said is that they are either few in number or very shy. And then we shall always be countered by the fellow who is positive he saw several the other night ! Such hypotheses are by their very nature invulnerable.

In contrast, the opposite scientific hypothesis that the *genus* Leprechaun does not exist, except possibly as a freak of natural development in isolated instances, is extremely vulnerable. Indisputable evidence of widespread leprechaun activity will entirely refute it, even if no actual leprechaun can be produced for examination. It is essential that this characteristic be present in any scientific hypothesis, so that the collection of sufficient opposition evidence can establish its falsity.

*Adoption of the null hypothesis.*

Applying these basic principles to the problem of " ulcer-cancer," can it be said that this conception has really been adequately demonstrated clinically, or even that it forms an essential entity in morbid anatomy ? Or have we perhaps accepted its existence with little more justification than our ancestors had in welcoming the leprechaun ? The very phraseology of the relevant literature—" the incidence of ulcer-cancer probably does not exceed $x$ per cent "—suggests that this may be the case, and that pathologists are themselves engaged in an attempt to depreciate its frequency of occurrence. It would seem, therefore, that a more profitable approach to the problem will be to attack it from the opposite direction, by the assumption of a " null hypothesis". In other words, to investigate the situation on the postulate that ulcer and cancer always occur independently in the stomach, in the aetiological sense, and that their occasional intimate association is entirely a matter of pure chance.

Failing the demonstration of " ulcer-cancer " as a necessary and unmistakable microscopical entity, such a null hypothesis could still be challenged by statistical evidence that the relative frequency of occurrence of associated ulcer and cancer is significantly greater than the hypothesis predicts. If the frequency of such associated lesions is so high that it is unlikely to have arisen by chance, the aetiological connection would be established, but even then this might only indicate that ulcer is a favoured site for the development of a carcinoma destined to arise in any case. However, since it is on the relative frequencies of these lesions that the null hypothesis must stand or fall, it is now important to survey the position statistically. And this is best carried out in general terms in order to avoid errors present in the conflicting pathological figures, and any profitless mathematical resolution of them.

In such an investigation, general figures of cancer incidence are obviously valueless. The only data worthy of consideration are those which have been verified pathologically, and the analysis must therefore be carried from the population at large up to the point where gastric specimens arrive at the pathologist. In the statistical model of the situation about to be constructed, it is assumed that the specimens eventually arriving at the pathologist are drawn from a population whose characteristics with respect to both gastric ulcer and carcinoma may be regarded as stable over a period of time covered by any particular analysis ; and that, by virtue both of its size and its autogenerative properties, the composition of this population remains substantially unaffected by the withdrawal of the specimens. It will also be assumed that ulcer and cancer occur quite independently.

*Development of the statistical model.*

In the population postulated there will be a fraction, U, of individuals with gastric ulcer and a fraction, C, with carcinoma of the stomach. The fractions without these lesions will therefore be $(1 - U)$ and $(1 - C)$ respectively, and the whole population will be assignable to four categories in accordance with the following schematic :

| Category. | Characteristics. | Fraction of total population. |
|---|---|---|
| 1 . | Having cancer but not ulcer . | $C(1 - U)$ |
| 2 . | Having ulcer but not cancer . | $U(1 - C)$ |
| 3 . | Having both ulcer and cancer . | $UC$ |
| 4 . | Having neither lesion . | $(1 - U)(1 - C)$ |

If it could be shown that the ratio $\frac{\mathbf{UC}}{\mathbf{U}}$ (which measures the incidence of cancer among those in the population who have ulcer) was significantly greater than the quantity **C** ; or that the ratio $\frac{\mathbf{UC}}{\mathbf{C}}$ (which measures the incidence of ulcer among those in the population who have cancer) was significantly greater than the quantity **U** ; then this might be evidence that gastric ulcer is a predisposing cause of cancer. But there is no known reliable estimate of the value of **U** ; whilst the value of **C**, though an approximate estimate of it might be obtained from the Registrar-General's returns, cannot be meaningly correlated with the particular sample of the population on which any particular series of pathological figures is based. These two ratios are the ones commonly quoted in the literature. Unfortunately, however, they are of little meaning, since stomachs resected for carcinoma are anything but an unbiased sample of all cases of gastric cancer, and the same is equally true in the case of gastric ulcer. Evidently something better than this simple approach is essential.

Let us therefore consider a model stomach, which for simplicity consists of only two compartments. It may be illustrated diagrammatically as follows :

| Pyloric Zone | Lesser Curvature |
|---|---|

The model has been deliberately restricted to these two regions as they constitute the main sites of origin of both ulcer and cancer, though there is no reason why a similar analysis should not be made, if desirable, using a model with more compartments. All lesions outside these chosen areas will be excluded from our following analysis. In the population at large there will be a fraction, $u$, of all ulcers which occur in the pyloric zone, and another fraction, $c$, of all cancers located in the same compartment of the model. The population at large will then fall into the categories illustrated in Fig. 1 with the frequencies stated, the left-hand compartments of the diagrams representing the pyloric zone and the right-hand ones the lesser curvature ; the two lower sections showing the position with respect to the relative frequencies of double lesions occurring in the same region, compared with double lesions which are sited in separate regions of the stomach. We shall refer to such double lesions occurring in the same region of the stomach as " amalgamated lesions " throughout the remainder of this paper, even if they are actually separate. Within this limited selection of the whole data evidence might be found of a tendency for co-existing ulcer and cancer lesions to become amalgamated in this sense more frequently than predicted by the null hypothesis, on which basis the expected value of the ratio of amalgamated lesions to non-amalgamated lesions is seen from Fig. 1 to be—

$$A_1 = \frac{uc\mathbf{UC} + (1-u)(1-c)\mathbf{UC}}{u(1-c)\mathbf{UC} + c(1-u)\mathbf{UC}},$$

which, on cancelling throughout by **UC**, becomes—

$$A_1 = \frac{uc + (1-u)(1-c)}{u(1-c) + c(1-u)}.$$

If, in practice, the observed value for this ratio were significantly greater than this expected value, it would indicate that ulcer was at least a favoured site for the development of a cancer, if not an actual aetiological factor in its development. It will be seen that this ratio has the statistical advantage that it does not include the imponderable quantities U and C, but depends only on the site distribution factors $u$ and $c$. It will be convenient in future to refer to this ratio $A_1$ as the *Amalgamation Ratio.*

| | | |
|---|---|---|
| | | $(1 - U)(1 - C)$ |
| Ulcer | | $uU(1 - C)$ |
| | Ulcer | $(1 - u)U(1 - C)$ |
| Cancer | | $c(1 - U)C$ |
| | Cancer | $(1 - c)(1 - U)C$ |
| Ulcer | Cancer | $u(1 - c)UC$ |
| Cancer | Ulcer | $c(1 - u)UC$ |
| Ulcer and Cancer | | $ucUC$ |
| | Ulcer and Cancer | $(1 - u)(1 - c)UC$ |

FIG. 1.

A further interesting ratio emerges from the study of Fig. 1, which has the same desirable characteristics of independence of U and C. This is the ratio of amalgamated lesions at the pylorus to amalgamated lesions at the lesser curve. The value for this ratio on the null hypothesis is—

$$D_1 = \frac{uc\mathbf{UC}}{(1-u)(1-c)\,\mathbf{UC}},$$

which reduces on cancelling by **UC** to—

$$D_1 = \frac{uc}{(1-u)(1-c)}.$$

It will be convenient to refer to this ratio as the *amalgamation distribution ratio.* Whereas the *amalgamation ratio* simply indicates whether the total amount of amalgamation is greater than the expected value on the null hypothesis, this second ratio shows whether amalgamation occurs preferentially in one or other of the regions.

*Effect of the selection chain.*

The model shown in Fig. 1 gives the expected distribution of all cases of ulcer and carcinoma into the various categories. If the null hypothesis is true, we should expect that a random collection of pathological specimens from the population at large would agree with the predicted ratios within the limits of sampling error. But, of course, pathological specimens are not collected at random, and it is therefore essential to consider in some detail the effect of the selection chain along which patients have to pass before their stomachs become pathological specimens.

The first group illustrated in the model shown in Fig. 1, those cases which have neither ulcer nor cancer, will, of course, be entirely missing from the pathologist's data.

Consider next the two categories of simple ulcer. Is it likely that ulcer of the pyloric canal will have the same probability of passing along the reference chain to the pathologist as ulcer at the lesser curve ? Prepyloric ulcer, in its uncomplicated form, is notoriously difficult to demonstrate radiologically but, once diagnosed with certainty, is usually operated on without delay, since so many of these lesions prove eventually to have been malignant since their onset. Benign ulcers at this site tend to give rise to early symptoms from the secondary pylorospasm which they cause ; yet they are often undetected until pyloric obstruction supervenes later, as the result of gradual stenosis, or unless acute perforation occurs. On the other hand, lesser curvature ulcer does not tend to produce such early symptoms, but is easier to display radiologically. Perforation is rarer, but haemorrhage more liable to occur at a relatively early stage of its development, thus aiding clinical diagnosis. Yet because they often heal under medical régime, there is frequently a significant time-lag before such lesions reach the surgeon, during which death may intervene from repeated haematemeses.

Consider now the two similar cancer categories. Pyloric carcinoma is probably diagnosed earlier, on the whole, than is cancer of the body of the stomach, owing to the relatively rapid development of obstructive symptoms. On the other hand, it is more often inoperable at laparotomy, and therefore is less likely to reach the pathologist as a specimen.

There is thus a complex selection mechanism operating along the whole of the reference chain, from the differential symptomatology in the patient through the medical practitioner or consulting physician to the surgeon. Only after all this does the stomach become a potential pathological specimen, the likelihood of which is again governed by the surgeon's technical skill, and the influence of this on his decision whether to operate and finally to resect. Moreover, still other factors may influence this selection of specimens, which will be different for each pathologist. The number of patients with gastric ulcer under observation by the medical side of the hospital will be one of these, for associated " ulcer-cancer " lesions will have a better chance of being diagnosed early if the chronic ulcer lesions are kept under continual scrutiny by the consultant physician rather than being referred back to their own family doctors for routine treatment. Further, the alteration in symptomatology resulting from the development of a secondary cancer in these cases is often relatively small in the early stages. Much depends on the vigilance and diagnostic skill of the physician concerned.

The almost universal tendency among reporters is to ignore these very important weightings of their figures, and to hope that in the end they will cancel each other out over a large series. Comparison of one series with another—or even the first and second part of the same observer's series—soon demonstrates that this does not happen. The fact is that each pathologist's series must be considered a unique set, not only because of his particular pathological criteria for " ulcer-cancer " but, perhaps even more, because his experience will include a unique set of weights in the data.

*Introduction of the weighting factors.*

It is now evident that the simple model shown in Fig. 1, which portrays the situation in the population at large, cannot be taken as an adequate picture of the expected proportions in any given series of pathological specimens. In Fig. 2 appropriate weighting factors have been introduced, which will take unique values for each individual pathologist in accordance with the varying nature of the particular selection chain along which his specimens have passed. The group of the population having neither ulcer nor cancer will be completely absent from such specimens and so will have weight zero. This is the only weight that the data of all pathologists will have in common.

It is now possible to restate symbolically the expected values for the *amalgamation ratio* and *amalgamation distribution ratio*, allowing for these weightings. It is important to realise that these new expressions are not valid for the population at large but only for a collection of pathological specimens, and this distinction will be emphasised by referring to them as observed ratios. On this new model, the *observed amalgamation ratio* will have an expected value, subject to sampling variations, of—

$$A_2 = \frac{w_7uc + w_8(1-u)(1-c)}{w_5u(1-c) + w_6c(1-u)}.$$

And the *observed amalgamation distribution ratio* will have an expected value, subject to sampling variations, of—

$$D_2 = \frac{w_7 uc}{w_8(1-u)(1-c)}.$$

The weighting complications are not yet ended, however. In the analysis so far, the expression " amalgamated lesions " has been used to imply no more than ulcer and cancer situated in the same region of the stomach, but all such combined lesions are not necessarily regarded by the pathologist as cases of " ulcer-cancer". Each will be judged on the personal criteria of the individual

| | | |
|---|---|---|
| | | $w_0(1-U)(1-C)$ |
| Ulcer | | $w_1uU(1-C)$ |
| | Ulcer | $w_2(1-u)U(1-C)$ |
| Cancer | | $w_3c(1-U)C$ |
| | Cancer | $w_4(1-c)(1-U)C$ |
| Ulcer | Cancer | $w_5u(1-c)UC$ |
| Cancer | Ulcer | $w_6c(1-u)UC$ |
| Ulcer and Cancer | | $w_7ucUC$ |
| | Ulcer and Cancer | $w_8(1-u)(1-c)UC$ |

FIG. 2.

pathologist concerned over the period in question, and only a proportion of them will eventually be considered to fall into this histological group. Let us suppose that, in each individual series, $k_1$ represents this fraction of all pyloric amalgamated lesions thus designated " ulcer-cancer," and that $k_2$ represents the corresponding fraction along the lesser curve. These may be different, and must therefore be allowed for by appropriate separate weightings in the calculated ratios. Applying them to $D_2$ the final expression now becomes—

$$D_3 = \frac{k_1 w_7 uc}{k_2 w_8 (1-u)(1-c)}.$$

It will be seen that this expression represents the expected value on the null hypothesis of the " *ulcer-cancer* " *distribution ratio* as assessed by the pathologist, on the basis that " ulcer-cancer " is a component of the amalgamated lesions occurring at each site.

Similarly the ratio of pathologically diagnosed " ulcer-cancer " to all other associated cancer and ulcer lesions, which do not fall into this precise histological group, is now represented for each individual series by the expression—

$$A_3 = \frac{k_1 w_7 uc + k_2 w_8 (1-u)(1-c)}{w_5 u(1-c) + w_6 c(1-u) + (1-k_1) w_7 uc + (1-k_2) w_8 (1-u)(1-c)}.$$

It will be observed that this ratio includes for the first time in its denominator all the amalgamated lesions which are not included in the pathologist's " ulcer-cancer " category, as well as associated lesions occurring in different regions of the stomach. For this reason it carries a different pathological significance to that expressed by the previously defined *amalgamation ratio.* It now represents the true " *ulcer-cancer* " *ratio* on the null hypothesis—this term being used on the understanding that the existence of " ulcer-cancer " has yet to be proved. Its entity must be demonstrated by showing that the expected values we have quoted for the critical ratios are significantly different from the observed values. It will be seen that these two ratios, which were originally adopted because they had the desirable property of independence of the imponderables U and C, are nevertheless very obscure collections of weights resulting from the reference chain.

*Practical application of the analysis.*

The application of these weightings to the ratios commonly quoted in the relevant literature shows that, instead of being relatively simple, these are in fact extremely obscure. The first of them, the fraction of ulcers observed to show malignancy, denoted by the symbols $\frac{\mathrm{UC}}{\mathrm{U}}$ in our present system of classification and usually taken to estimate that ratio, is actually an estimate of the much more complex quantity,

$$\frac{k_1 w_7 uc\mathrm{UC} + k_2 w_8 (1-u)(1-c)\mathrm{UC}}{w_1 u\mathrm{U}(1-\mathrm{C}) + w_2(1-u)\mathrm{U}(1-\mathrm{C}) + w_5 u(1-c)\mathrm{UC} + w_6 c(1-u)\mathrm{UC} + w_7 uc\mathrm{UC} + w_8(1-u)(1-c)\mathrm{UC}},$$

whilst the second commonly quoted ratio, the fraction of all cancers observed to originate in ulcer, which is generally supposed to be represented by the ratio $\frac{\text{UC}}{\text{C}}$, is equally complex and is in fact—

$$\frac{k_1 w_7 uc\text{UC} + k_2 w_8 (1-u)(1-c)\,\text{UC}}{w_3 c(1-\text{U})\,\text{C} + w_4(1-c)(1-\text{U})\,\text{C} + w_5 u(1-c)\,\text{UC} + w_6 c(1-u)\,\text{UC} + w_7 uc\text{UC} + w_8(1-u)(1-c)\,\text{UC}.}$$

These ratios are even more obscure than those giving the values for $A_3$ and $D_3$, since there is no way of eliminating from them the additional quantities U and C. Each of them contains no less than twelve unknown weights, so that it is obvious that little reliance can be placed on estimates of the incidence of " ulcer-cancer " obtained in this way. Even if approximations could be made in respect of the other unknowns, the basic imponderables U and C still present a problem difficult of solution. The value of C might reasonably be estimated approximately, as has already been pointed out, from the Registrar-General's figures and the computable expectation of life with cancer of the stomach ; but no reliable estimate of U is available. Doll and Avery Jones (1951) have essayed this problem, realising the importance of obtaining some reliable approximation of it, but their results are open to criticism (Editorial, *Brit. med. J.*, 1951).

In view of the intractability of this approach, let us consider the somewhat simpler ratios $A_3$ and $D_3$, both of which suggest alternative modes of attack on the problem. The " *Ulcer-Cancer* " *Ratio*, $A_3$, although independent of both U and C, still contains 8 unknowns. Moreover, 2 of them, $w_5$ and $w_6$, are the weightings for referability of associated ulcer and cancer lesions in separate compartments of the same stomach—the pathological data for the frequency of which is scanty and its clinical recognition difficult. The " *ulcer-cancer* " *distribution ratio* $D_3$ is, however, more promising in that it includes only 6 unknowns. Essentially, this expression—

$$D_3 = \frac{k_1 w_7 uc}{k_2 w_8 (1-u)(1-c)}$$

is seen to be

$$= \left[\frac{k_1}{k_2}\right] \times D_2,$$

and it should be capable of detecting any significant aetiological factor between ulcer and cancer, conducing to the preferential choice of zones for development.

Despite the microscopical difficulties sometimes experienced in determining accurately the precise starting-point of pyloric growths, which may occasionally influence the opinion whether the lesion is an " ulcer-cancer " or merely an amalgamated one without aetiological connotation, it is probably true that any individual pathologist's criteria will be independent of the site of origin. If so, then $k_1 = k_2$ and the null hypothesis expectation for $D_3$ will be identical with that for $D_2$. Either of these two expressions the " *ulcer-cancer* " *distribution ratio* or the *observed amalgamation distribution ratio*, may therefore be used for testing the truth of this hypothesis. In both instances the same 4 unknowns are involved—$u$, $c$, $w_7$ and $w_8$—though these can be reduced mathematically to 3, since only the ratio $\frac{w_7}{w_8}$ of the 2 weights is required and not their separate explicit values. If the individual pathologist could be given this ratio of the weights concerned, and the values for $u$ and $c$, he could then calculate the expected values for $D_2$ and $D_3$, against which the observed values for these ratios in his own series could be tested.

The evaluation of $u$ and $c$ from existing figures is, however, rendered impossible by the effect of the reference chain and the different criteria adopted for the boundaries of the various stomach zones, nearly all the published work on stomach pathology conflicting in this respect. Minor differences on the latter score between different series may be partially taken up in the weights $w_7$ and $w_8$, since all comparisons will be internal ones within each set of individual data, but the question is of academic interest only so long as marked reference chain effects persist. Separate independent research, deliberately designed to eliminate the effect of reference chains, would be necessary for their estimation. Moreover, even if the individual pathologist was supplied with values for $u$ and $c$, he would still be unable to determine whether his observed values for $D_2$ and $D_3$ differed from the null hypothesis expectation for these ratios. The ratio $\frac{w_7}{w_8}$ is inherently a property of his own series, representing the effect of the reference chain operating in his particular case. It can therefore only be determined by using his own observed frequencies for $u$ and $c$ and checking them against those established through such an independent survey. Manifestly, he cannot use his own data both to establish the expected value of the expressions $D_2$ and $D_3$, and then to see if his observed values differ from expectation. The position, from the point of view of the individual pathologist, is that significant departures in this respect from the null hypothesis predictions are inextricably confused with the effect of the reference chain ; and the same applies, even more, to the ratios $A_2$ and $A_3$, which are under the influence of the reference chain to a greater extent.

This is a point of paramount importance. Its implication is that no pathologist, however competent, and no consensus of opinion among pathologists, however widespread, can refute the null hypothesis on the basis of existing figures.

It follows inevitably that the solution to this problem of the incidence of "ulcer-cancer" must await a research project specifically designed to remove the insuperable obstacle facing the individual pathologist—the effect of the reference chain represented by the weights $w$ in our analysis. The results of such an investigation could then be evaluated by means of the ratios $A_1$ and $D_1$, which only involve the site distribution factors $u$ and $c$. It would be essential for this purpose to establish some sufficiently accurate convention defining the boundaries between the various stomach zones. Admittedly, these are not always easy to demarcate in practice, but the contrivance of some effective standard in this respect ought not to present insuperable difficulties.

The most hopeful way of establishing reliable values for $u$ and $c$ would appear to be by planned, clinical research at a number of large centres. The assiduous co-operation of all the local general practitioners surrounding these centres would be an essential, so that every case of a suspected stomach lesion reaches them at the earliest possible moment. By avoiding the symptomatic treatment of such patients for prolonged periods at home before reference for consultative opinion, accurate site diagnosis of the lesion would be rendered more certain. There would also be less chance of gastric ulcers healing undiagnosed ; and less possibility of secondary carcinoma, which may have arisen at or near the site of a prior ulcer, developing sufficiently to obscure the primary pathology. Every patient reaching such a centre would have to be most carefully investigated and kept under constant review, radiological and gastroscopic checks being repeated at adequate intervals. All cases of proven gastric ulcer would have to be followed up in the out-patient department until healing was complete, and any failing to respond to medical treatment, together with every case of suspected neoplasm, pursued vigilantly to either theatre or post-mortem room. Similarly, all patients admitted to such centres as emergencies, with complications such as haematemesis, perforation or acute exacerbation of an ulcer, would have to be transferred to the out-patient department for further review after their discharge.

Projected clinical research of this type would, of course, besides entailing the wholehearted assistance of the neighbouring general practitioners, require a great volume of routine follow-up work and the effective co-operation of the patients concerned. But, only by such measures, can the diagnostic net be thrown wide enough to be able to detect the presence of a secondary neoplasm adjacent to the gastric lesion at the critical moment for accurate diagnosis. The research would have to be based on several centres, whose respective findings could finally be checked for statistical homogeneity, and these centres ought to be carefully selected. They should be situated at large-population centres which are growing, to minimise loss of patients during prolonged observation from drift to other areas ; and in provincial towns rather than in very large cities, where it might be difficult to trace their admission to, or treatment at, a large number of alternative hospitals for a gastric emergency or some intercurrent disease.

If the establishment of such centres were practicable, it might be possible to eliminate the reference chain weights $w$ for all practical purposes, and thus arrive at reliable values for the site distribution factors $u$ and $c$. There are, of course, dangers of leakage in the procedure outlined. In addition to the inevitable loss of some patients during the prolonged observation necessary, there is the certainty that some cases would not reach autopsy. Moreover, there will be a fraction of the population who will not seek any medical advice until the disease has progressed too far for any statistical value to be elicited regarding the site diagnosis. This is not the place to discuss the details of such an investigation, but the essential problem regarding such losses is not so much their magnitude as whether they are likely to introduce bias in the determination of $u$ and $c$.

If unbiased estimates of $u$ and $c$ could be obtained in this way, the data collected would suffice to test the null hypothesis expectations. Cases with single lesions could be used to provide values for these quantities, and thus for the expected values of $A_1$ and $D_1$, of a high degree of accuracy deriving from the large number of observations on which they would be based. Cases with double lesions would then give an adequate check on such results, using the incidence figures of ulcer and cancer in different stomach zones for this purpose in addition to those of amalgamated lesions in the same zone. As such a challenge to the null hypothesis involves absolutely no assumption of the pathological criteria for " ulcer-cancer ", the establishment of this entity by so direct a statistical investigation would be highly significant and the direction of departure from the null hypothesis expectations by both amalgamated and separate lesions would be most instructive. It would, in any case, still be open to the pathologists at the research centres, providing that they can agree the criteria for the establishment of this entity, to continue to make diagnoses of " ulcer-cancer " and, on the assumption that $k_1 = k_2$, to dispute the null hypothesis in terms of the *" ulcer-cancer " distribution ratio*, this expression—

$$D_3 = \frac{uc}{(1 - u)(1 - c)},$$

now having the same expected value on the null hypothesis as $D_1$, once the effect of the weights has been removed, though its actual significance is different.

### CONCLUSIONS.

By investigating this problem along the lines suggested, it ought to be possible eventually to accumulate sufficient evidence to confirm or refute the null hypothesis that " ulcer-cancer " is not a true entity. Moreover, if it should be shown that this lesion is indeed a pathological actuality, it should also be possible to determine a reasonable approximation of its incidence in a statistically sound manner—an impossible task on the existing evidence. Clinicians would then no longer be condemned to wander in a maze of conflicting pathological evidence regarding the ultimate prognosis of chronic gastric ulcer, but would be given an answer which would assist them materially in their treatment of this disease. Until that time comes, however, it seems that there is no more cogent basis for the assumption that malignant degeneration is a common event in gastric ulcer than argument by analogy from carcinomatous changes consequent on chronic inflammation elsewhere in the body.

### SUMMARY.

1. An investigation has been made into the existing evidence for secondary neoplastic change in chronic gastric ulcer.

2. Insufficient purely clinical data exists to justify the frequent diagnosis of " ulcer-cancer " in its accepted connotation, and until pathologists in general can agree their criteria for the microscopical establishment of this entity, the histological evidence for such malignant change cannot be accepted at face value.

3. Furthermore, it is suggested that this concept of " ulcer-cancer " as a not uncommon sequel to longstanding gastric ulceration is equivocal, probably having arisen initially by analogy with similar transformation in chronic inflammatory tissue elsewhere in the body.

4. Since a dialectic approach of this kind may lead to most fallacious conclusions, this investigation has accordingly been based on the postulate of a null hypothesis, to the effect that " ulcer-cancer " does not exist as a true entity in the aetiological sense.

5. The construction of a statistical model of the problem on these lines shows, however, that the figures commonly quoted in the literature for the incidence of " ulcer-cancer " are invalid and illusory. So many imponderables are concerned that attempts to calculate such ratios on the basis of existing data must be foredoomed to failure.

6. A new approach to the problem is therefore suggested, designed to remove the effects of these unknowns. It entails the early reference to special research centres of all patients with symptoms suggestive of any gastric pathology for careful investigation and subsequent observation over an adequate period of time, and should be capable of yielding figures of definite statistical value.

### REFERENCES.

BROWN, R. C.—(1945) "Ulcer of the Stomach, Duodenum and Jejunum" 'Oxford Medicine.' London (Oxford University Press), Vol. 3, Chap. III.

DOLL, R., AND AVERY JONES, F.—(1951) *Spec. Rep. Ser. med. Res. Coun. Lond.*, No. 276.

Editorial.—(1951) *Brit. med. J.*, i, 463.

EUSTERMAN, G. B., AND BALFOUR, D. C.—(1936) 'The Stomach and Duodenum.' Philadelphia (W. B. Saunders Co.).

EWING, J.—(1940) 'Neoplastic Diseases: A Treatise on Tumours.' Philadelphia (W. B. Saunders Co.).

HAUSER, G.—(1926) 'Handbuch der speziellen pathologischen Anatomie und Histologie.' Berlin (Julius Springer), Vol. 4, Part I, p. 339.

HURST, A. F., AND STEWART, M. J.—(1929) 'Gastric and Duodenal Ulcer.' London (Oxford University Press).

IHRE, B. J. E., AND MÜLLER, R.—(1943) *Acta med. scand.*, **116**, 33.

IVY, A. C., GROSSMAN, M. I., AND BACHRACH, W. H.—(1950) 'Peptic Ulcer.' Philadelphia (Blakiston Co.).

LIVINGSTONE, E. M., AND PACK, G. T.—(1939) 'End Results in Treatment of Gastric Cancer.' New York (Paul B. Hoeber Inc.).

MARSHALL, S. F.—(1944) *Surg. Clin. N. Amer.*, **24**, 618.

MARTIN, C. F.—(1909) "Organic Diseases of the Stomach." In: Osler's & McCrae's *System of Medicine*, **5**, 175.

STEWART, M. J.—(1926) *J. Path. Bact.*, **29**, 321.

UDAONDO, C. B.—(1939) "Relaciones entre la úlcera y el cáncer gastrico," *Arch. argent. Enferm. Apar. dig.*, **14**, 1.